# Co-delivery of Dual Toll-Like Receptor Agonists and Antigen in Poly(Lactic-Co-Glycolic) Acid/Polyethylenimine Cationic Hybrid Nanoparticles Promote Efficient *In Vivo* Immune Responses

**DOI:** 10.3389/fimmu.2017.01077

**Published:** 2017-09-13

**Authors:** Mahboubeh Ebrahimian, Maryam Hashemi, Mohsen Maleki, Gholamreza Hashemitabar, Khalil Abnous, Mohammad Ramezani, Alireza Haghparast

**Affiliations:** ^1^Division of Biotechnology, Faculty of Veterinary Medicine, Ferdowsi University of Mashhad, Mashhad, Iran; ^2^Immunology Section, Faculty of Veterinary Medicine, Ferdowsi University of Mashhad, Mashhad, Iran; ^3^Nanotechnology Research Center, School of Pharmacy, Mashhad University of Medical Sciences, Mashhad, Iran; ^4^Department of Pathobiology, Faculty of Veterinary Medicine, Ferdowsi University of Mashhad, Mashhad, Iran; ^5^Pharmaceutical Research Center, Mashhad University of Medical Sciences, Mashhad, Iran

**Keywords:** adjuvants, CpG ODN, monophosphoryl lipid A, poly(lactic-co-glycolic) acid nanoparticles, polyethylenimine, resiquimod, toll-like receptor agonist, vaccine

## Abstract

Strategies to design delivery vehicles are critical in modern vaccine-adjuvant development. Nanoparticles (NPs) encapsulating antigen(s) and adjuvant(s) are promising vehicles to deliver antigen(s) and adjuvant(s) to antigen-presenting cells (APCs), allowing optimal immune responses against a specific pathogen. In this study, we developed a novel adjuvant delivery approach for induction of efficient *in vivo* immune responses. Polyethylenimine (PEI) was physically conjugated to poly(lactic-co-glycolic) acid (PLGA) to form PLGA/PEI NPs. This complex was encapsulated with resiquimod (R848) as toll-like receptor (TLR) 7/8 agonist, or monophosphoryl lipid A (MPLA) as TLR4 agonist and co-assembled with cytosine–phosphorothioate–guanine oligodeoxynucleotide (CpG ODN) as TLR9 agonist to form a tripartite formulation [two TLR agonists (inside and outside NPs) and PLGA/PEI NPs as delivery system]. The physicochemical characteristics, cytotoxicity and cellular uptake of these synthesized delivery vehicles were investigated. Cellular viability test revealed no pronounced cytotoxicity as well as increased cellular uptake compared to control groups in murine macrophage cells (J774 cell line). In the next step, PLGA (MPLA or R848)/PEI (CpG ODN) were co-delivered with ovalbumin (OVA) encapsulated into PLGA NPs to enhance the induction of immune responses. The immunogenicity properties of these co-delivery formulations were examined *in vivo* by evaluating the cytokine (IFN-γ, IL-4, and IL-1β) secretion and antibody (IgG1, IgG2a) production. Robust and efficient immune responses were achieved after *in vivo* administration of PLGA (MPLA or R848)/PEI (CpG ODN) co-delivered with OVA encapsulated in PLGA NPs in BALB/c mice. Our results demonstrate a rational design of using dual TLR agonists in a context-dependent manner for efficient nanoparticulate adjuvant-vaccine development.

**Graphical Abstract:**
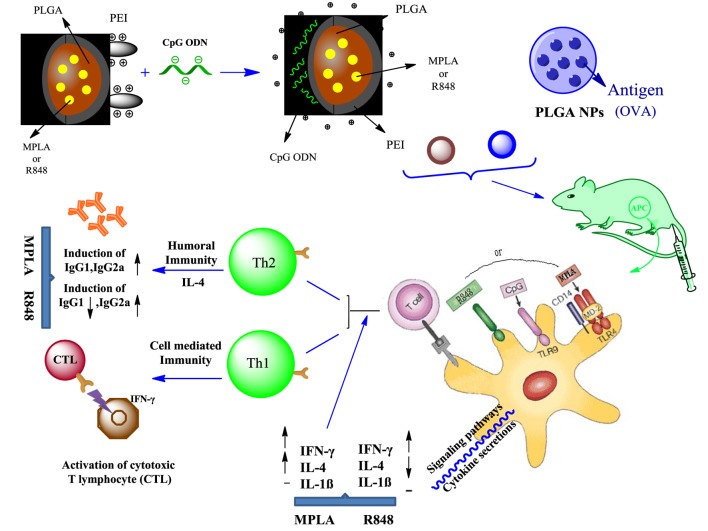
PEI was physically conjugated to PLGA NPs to form PLGA/PEI NPs. This complex was encapsulated with R848 or MPLA and co-assembled with CpG ODN on the surface of NPs to form a tripartite complex [two TLR agonists (inside and outside NPs) and PLGA/PEI NPs as delivery system]. The immunogenicity properties of these co-delivery formulations were examined *in vivo* by evaluating the cytokines secretion and antibody production.

## Introduction

The recent advances in vaccine development have moved the area from traditional vaccines using whole microorganisms to subunit vaccines containing only purified or modified antigenic proteins. Low immunogenic potential of such vaccines compared to live attenuated pathogens has motivated the research toward developing new adjuvants with biomimetic potentials to promote robust and effective innate and adaptive immune responses. Pattern recognition receptors (PRRs) are the main class of innate immunity sensors, recognizing diverse sets of pathogen-associated molecular patterns (PAMPs) and considered to be the target of novel molecular adjuvant developments. One of the major families of PRRs, Toll-like receptors (TLRs) are expressed by a variety of cells and capable of inducing innate immune responses and initiate the pathways toward effective adaptive immune responses. During natural infection, most pathogens encounter with the immune system through multiple danger signals including PAMPs to stimulate multiple PRRs, resulting in a synergistic upregulation of pro-inflammatory cytokines and chemokines and subsequent activation of antigen-presenting cells (APCs). Therefore, the combination of multiple PRRs agonists and proper delivery systems may be a promising strategy in any artificial immunization approach to induce effective immune responses in the context of vaccine-adjuvant development (1–4).

There is strong evidence suggesting that engagement of TLRs with PAMPs results in the skewing of T helper (Th) immune responses toward either Th1 or Th2 cytokine profiles (5–7).

Optimal vaccine design requires antigen(s), adjuvant(s), and a vehicle, in which co-administration of antigen and adjuvant will be delivered by a biocompatible vehicle to sentinel dendritic cells (DCs) in draining lymph nodes, promoting the development of effective immune responses (8). Recently, nanoparticles (NPs) have emerged as an attractive vehicle for synchronized targeted delivery of antigens and adjuvants to the immune system. NPs prepared from the biodegradable and biocompatible polymer, poly (lactic-co-glycolic acid) (PLGA), have extensively been used in clinical settings for drug delivery and are currently the subject of intensive investigation as antigen and adjuvant delivery system for vaccine purposes (9, 10). Synergistic activation of cytokine production in human and mouse DCs by combinations of TLRs ligands has been demonstrated *in vitro* (11). Such synergies may also be relevant for *in vivo* responses to vaccination. For instance, in non-human primates, combinations of TLR7/8 and TLR9 agonists enhanced the induction of neutralizing antibody titers against a human immunodeficiency virus envelope glycoprotein (12). When Rhesus macaques were immunized with PLGA NPs encapsulating TLR4 and TLR7/8 agonists mixed with two soluble recombinant antigens of simian immunodeficiency virus, vaccine containing PLGA NPs delivering dual TLRs agonists (TLR4 and TLR7/8) induced robust innate as well as antigen-specific antibody immune responses, which was greater in magnitude and persistence, and enhanced plasmablast responses compared to those achieved with aluminum hydroxide (alum)-adjuvanted vaccine (13). Encapsulating TLR4 and TLR7 agonists in PLGA NPs was found to induce a synergistically high antibody titers and increase the number of germinal centers in lymph nodes following vaccination in mice, while preventing the toxic side effects of the free adjuvant compounds (14). Further studies have shown that the delivery of TLRs agonists by PLGA NPs promotes the induction of protective and therapeutic immune responses against diseases such as leishmaniasis (15), hepatitis B (16), West Nile Encephalitis (17), avian influenza (18), and cancer (19–21). These studies highlight the potential of PLGA NP-encapsulated TLR ligands as vaccine adjuvants.

Therefore, in the present study we sought to construct and evaluate a PLGA NP-based adjuvant delivery platform using unmethylated cytosine–phosphorothioate–guanine oligodeoxynucleotide (CpG ODN) as TLR9 agonist, monophosphoryl lipid A (MPLA), a TLR4 agonist, and clinically approved Th1 polarizing adjuvant which is 100–10,000 times less toxic than lipopolysaccharide (LPS) (22) and resiquimod (R848) as TLR7/8 agonist. Additionally, we sought to enhance the co-delivery of MPLA or R848-encapsulated PLGA NPs along with CpG ODN in one platform using polyethylenimine (PEI), a cationic polymer which has previously been shown to increase the magnitude of the adaptive immune response with a shift toward Th1 response (23, 24).

Polyethylenimine was physically conjugated to PLGA NPs to form PLGA/PEI NPs. This complex was encapsulated with R848 or MPLA and co-assembled with CpG ODN on the surface of NPs to form a tripartite complex [two TLR agonists (inside and outside NPs) and PLGA/PEI NPs as delivery system]. Cellular toxicity assay was performed with murine macrophage cells (J774 cell line) for evaluating the cytotoxicity of cationic PLGA/PEI NPs. The uptake capacity of cationic NPs containing dual adjuvant CpG ODN and either MPLA or R848 was evaluated using FITC-labeled PEIs. Ovalbumin (OVA)-encapsulated PLGA NPs as model antigen and alum as standard adjuvant were used as control immunization. The immunogenic potentials of these multiple NP adjuvant formulations were examined *in vivo* by evaluating the cytokine secretion (IFN-γ, IL-4, and IL-1β) and antibody production (IgG1 and IgG2a). The results demonstrated enhanced and robust Th1/Th2 immune responses elicited by co-delivery of dual TLRs agonist in PLGA/PEI NPs.

## Materials and Methods

### Materials

Poly (lactic-co-glycolic acid) (50:50, Resomer^®^ RG 502H), polyvinyl alcohol (PVA; MW 31,000–50,000), and albumin from chicken egg white (OVA) grade VI were purchased from Sigma-Aldrich (Munich, Germany). Type C CpG ODN 2395 as TLR9 agonist was purchased from Bioneer (Daejeon, Korea). Synthetic MPLA as TLR4 ligand and imidazoquinoline compound (R848) as TLR7/8 ligand were purchased from InvivoGen (San Diego, CA, USA). Branched PEI (average MW 10 kDa) was purchased from Polyscience, Inc. (Warrington, FL, USA). Ethidium bromide was obtained from Cinnagen (Tehran, Iran). Spectra/Por dialysis membranes were purchased from Spectrum Laboratories (Houston, TX, USA). Cell Titer 961 aqueous one solution cell proliferation assay (MTT) was obtained from Promega (Madison, WI, USA). All other reagents were of analytical grade and received from commercial sources.

### Cell Culture

J774 (murine macrophage) cell line was purchased from Pasteur Institute of Iran and cultured at 37°C, 95% humidity, and 5% CO_2_ atmosphere in DMEM high glucose medium supplemented with 1% l-glutamine (2 mM), 100 µg/ml streptomycin, 100 units/ml penicillin, and 10% fetal bovine serum (FBS; Gibco, NY, USA).

### Synthesis of NPs

#### Preparation of OVA, R848, and MPLA-Loaded PLGA NPs

Poly(lactic-co-glycolic) acid nanoparticles containing OVA were prepared by double emulsion-solvent evaporation technique (w/o/w) with some modifications. Briefly, 200 µl OVA (20 mg/ml) was added to 8% w/v PLGA in 1 ml of dichloromethane/acetone mixture (ratio 1:4) under stirring at 1,300 rpm and then the mixture was sonicated (Ultrasonic processor 200H) at 80% amplitude for 1 min on ice. Next, first emulsion was added to 4 ml of an aqueous solution of PVA (5%) under sonication for 10 min on ice to form w/o/w double emulsion. Subsequently, the resulting emulsion (w/o/w) was added to 30 ml of an aqueous solution of 0.1% PVA and vigorously stirred overnight at room temperature to evaporate organic solvent. The PLGA NPs were then centrifuged at 20,000 rpm at 4°C for 20 min and washed three times with deionized water followed by lyophilization (TAITEC Corporation, Japan). NPs were stored at 4°C for later uses. PLGA NPs containing R848 and MPLA were also prepared as above except that 5% w/v PLGA in organic solvent along with 200 and 70 µg of TLR ligands were used, respectively. Empty PLGA NPs were also prepared as control.

#### Preparation of PLGA/PEI_10k_ Tripartite Formulation

Polyethylenimine aqueous solution (1 mg/ml) was added to the aforementioned PLGA NPs suspension containing either R848 or MPLA and incubated 30 min at room temperature. After this period, CpG ODN was added and incubated for 20 min at room temperature in order to form the cationic NPs (polyplexes).

### NP Characterization

#### Particle Size and Zeta Potential

The hydrodynamic diameter of synthetized NPs was assessed by DLS on a Zetasizer Nano ZS (Malvern Instruments, Malvern, UK). Three independent measurements were performed to generate the intensity-based size distribution profile.

#### Polyplex Formation between CpG ODN and Cationic Co-polymer

Polyplexes were prepared by adding various concentrations of PEI in PLGA NP formulations in *N*-(2-hydroxyethyl) piperazine-*N*-(2-ethanesulfonic acid) (HEPES) buffer to equal volume of buffer containing constant amount of 400 ng CpG ODN. After incubating for 20 min at room temperature, polyplexes were formed at a range of carrier/CpG ODN ratios (C/P).

#### Agarose Gel Retardation Assay

Retardation of CpG ODN mobility by cationic NPs was evaluated by agarose gel retardation assay. The polyplex solution was first prepared under a predetermined C/P ratio with 400 ng CpG ODN in HEPES buffer. Then, the polyplex solution was loaded onto 1% agarose gel (w/v) containing ethidium bromide (0.5 µg/ml) in TAE buffer (pH 7.4). The naked CpG ODN was used as control. The gel was run under 80 V for 30 min, and CpG ODN migration was recorded on a UV transilluminator system (Uvidoc, Cambridge, UK). DNA ladder (ThermoFisher Scientific, Carlsbad, CA, USA) was used as DNA size marker.

#### Determination of Encapsulation Efficiency (EE%) of OVA, R848, and MPLA in PLGA NPs

To determine the amount of encapsulated agents (OVA, MPLA, and R848) in PLGA, PLGA NPs (5 mg) were treated with 0.1 M NaOH solution overnight to break the NPs, followed by neutralizing with 1 M HCL and spinning down at 14,000 rpm for 2 min. After that, supernatant was collected and each sample was assessed in triplicate using BCA assay (562 nm), UV-VIS spectrophotometry (327 nm) and fluorimetry (551–567 nm) for OVA, R848, and MPLA, respectively. EE% and loading content (LC%) of PLGA NPs were calculated by the following equations:

$$\text{EE}\left(\text{\%}\right)=\frac{\text{Amount of cargo in NPs}}{\text{Amount of cargo used for en​capsulation}}\times 100$$

$$\text{LC}\left(\text{\%}\right)\text{=}\frac{\text{Mass of cargo in NPs}}{\text{Mass of NPs}}\times \text{100.}$$

#### Structural Characterization

Morphology of PLGA/PEI NPs was examined by field emission scanning electron microscopy (SEM) (FE-SEM, Mira IIIFEG, TESCAN-UK, Ltd). The sample solution in deionized water (1 mg/ml) was dehydrated on a metal stub for FE-SEM analysis. The morphology and bilayer configuration of PLGA/PEI co-polymer were also investigated by transmission electron microscopy (TEM) (Leo 912 AB, Carl Zeiss, Germany).

### *In Vitro* Study

#### *In Vitro* Release Profile of OVA, R848, and MPLA from PLGA NPs

*In vitro* release of OVA, R848, and MPLA from PLGA NPs in PBS at 37°C was evaluated over a period of 10 days. NPs containing OVA, R848, and/or MPLA (5 mg) were dispersed in 5 ml of PBS (pH 7.4). The samples were incubated at 37°C on shaker (90 rpm) and at predetermined intervals (1, 2, 3, 4, and 5 h and 1, 2, 3, 4, till 10 days), tubes were centrifuged at 3,000 × g for 10 min. In the supernatants, the amount of OVA, R848, and MPLA released from the particles were determined by BCA assay, UV-VIS spectrophotometry, and fluorimetry, respectively. Meanwhile, removed supernatants were replaced with the same amount of fresh PBS to keep the medium volume constant. Release data were expressed as the cumulative percentage of OVA, R848, and MPLA in comparison with the initial content of these molecules in the NPs versus time. Each independent experiment was done in triplicates for each formulation, and experiments were repeated at least thrice.

#### Assessment of Vector-CpG ODN Complex Stability

To assess the stability of the vector–oligonucleotide complex, we studied the release of the CpG ODN from the complex for 7 days at 37°C using gel electrophoresis as described in previous step.

#### Cytotoxicity Assay

MTT assay was performed with J774 cells for evaluating the cytotoxicity of cationic PLGA/PEI NPs. J774 cells were seeded into a 96-well microplate at 10^4^ cells per well, cultured in 37°C, 95% humidity, and 5% CO_2_ for 24 h in 100 µl DMEM medium containing high glucose and 10% FBS. Thereafter, PLGA/PEI polyplexes (C/P ratio 2–6) were individually placed into the wells and further incubated for another 48 h. Subsequently, 20 µl of MTT reagent (0.5 mg/ml in phosphate buffer 0.1 M pH 7.4) was added into each well and incubated for 4 h. After removing the medium, DMSO (100 μl/well) was added and shaken for 10 min to dissolve formazan. Each sample with three replicates was analyzed on a microplate reader at wavelengths of A570 and A630 (Infinite NanoQuant M200, Tecan, Switzerland). PEI 25 kDa at C/P 0.8 was used as the reference for the MTT cytotoxicity assay.

### Uptake Study

#### Uptake Study on J774 Cells Treated with Cationic NPs Containing CpG ODN

To evaluate the uptake capacity of cationic NPs containing dual adjuvant CpG ODN and either MPLA or R848, the FITC-labeled PEIs were used. J774 macrophage cells were seeded in 24-well plates at 4 × 10^4^ cells/well and cultured overnight. 100 µg of either unmodified PEI/CpG ODN or PLGA/FITC-PEI/CpG ODN NPs were added to each wells containing fresh medium. FITC-PEI 25 kDa at C/P 0.8 and untreated cells were used as positive and negative controls, respectively. Cells were incubated for 48 h and then evaluated by FACS analysis. Propidium iodide gating was used to eliminate dead cells, and 10,000 total events were collected for analysis.

### *In Vivo* Study

#### Immunization of Mice

Female BALB/c mice (8–12 weeks) were purchased from Pasteur Institute (Tehran, Iran) and kept according to the ethical statement. Groups of 14 mice (*N* = 3) (Table 1) were immunized twice (days 0 and 21) by the subcutaneous (S.C.) injection at tail base. For each injection, mice received 200 µl of either sample or physiological saline (as control group). One week after the second vaccination, spleens were taken from mice and splenocytes were isolated and used in ELISpot assay for cytokine detection. The collected sera were kept in −80°C for enzyme-linked immunosorbent assay (ELISA) assay.

**Table 1:** *In vivo* immunization of mice by core–shell PLGA/PEI NPs containing either single or dual TLR agonists.

Group	Delivery systems	Antigen (μg) (OVA)	Adjuvant (μg)			
			CpG ODN	MPLA	R848	Alum
1	PEI, PLGA	10	10	–	–	–
2	PEI, PLGA	10	10	3	–	–
3	PEI, PLGA	10	10	–	4	–
4	PLGA	–	–	3	–	–
5	PLGA	–	–	–	4	–
6	PEI	–	10	–	–	–
7	–	–	10	–	–	–
8	–	10	–	–	–	–
9	–	10	–	–	–	100
10	–	–	–	–	–	–
11	PLGA	–	–	–	–	–
12	PLGA	10	–	–	–	–
13	–	–	–	3	–	–
14	–	–	–	–	4	–

*Alum, aluminum hydroxide; CpG ODN, cytosine–phosphorothioate–guanine oligodeoxynucleotide; MPLA, monophosphoryl lipid A; NPs, nanoparticles; OVA, ovalbumin; PEI, polyethylenimine; PLGA, poly(lactic-co-glycolic) acid; TLR, toll-like receptor*.

#### Measurement of Antibody Isotypes Titers in Serum

Serum obtained from mice was analyzed for IgG1 and IgG2a antibody titers. All samples were tested by ELISA kits (e-Bioscience, Vienna, Austria) according to the manufacturer’s instructions, using 96-well polystyrene plates (Corning costar 9018, flat bottom). Briefly, plates were filled with 100 µl capture antibody diluted in coating buffer at 4°C overnight and washed twice with Tween 20 (0.05% v/v) containing PBS (pH 7.4). To prevent non-specific binding to the antibody, a blocking step was performed using blocking buffer. The samples diluted in PBS (1/10,000) and added to the plates which were then incubated for 2 h at room temperature and washed thoroughly as before. HRP-conjugated anti-mouse IgG (subtypes IgG1 and IgG2a) (100 µl) was then added to each well, and the plates were again incubated for 3 h at room temperature on microplate shaker at 400 rpm, followed by washing as before. Enzyme substrate, tetramethylbenzidine (100 µl) was added to each well and the reaction was stopped after 15 min with 50 µl 2 N H_2_SO_4_. The optical density at 450 nm using 570 nm filter as a reference wavelength was read in a microplate reader (Infinite NanoQuant M200, Tecan, Switzerland).

#### Detection of Cytokines by ELISpot and ELISA

Naive and immunized mice were sacrificed by cervical dislocation at day 7 after secondary immunization. The spleens were removed and placed in RPMI 1640 media (Gibco-BRL, UK) under sterile conditions. Each spleen was chopped, and cells within empirical groups were pooled in one tube. The cellular suspension was centrifuged at 800 × *g* for 10 min, supernatant was discarded, and the pellet was washed twice with PBS. Then, the splenocytes were suspended in ACK lysis buffer for 2 min and replenished with RPMI 1640 to stop the erythrocyte elimination reactions. This suspension was centrifuged (800 × *g*, 5 min), and the pellet was resuspended in fresh complete RPMI medium. 5 × 10^4^ cells were added to 96-well MultiScreen-IP, clear styrene plates (MAIPS 4510, Millipore, Ireland) along with antigen (10 µg) added in a final volume 200 µl per well. Negative (wells without antigen) and positive [wells containing ConA (2 µg/ml)] wells were used as controls. Plates were incubated at 37°C, 95% humidity, and 5% CO_2_ for 24 h, and IFN-γ- and IL-4-producing splenocytes were determined using a commercial ELISpot Ready-SET-Go kit according to the manufacturer’s instruction (eBioscience, Vienna, Austria). When spots appeared, counting was done by Kodak 1D image analysis software Version 3.5 (Eastman Kodak, Rochester, NY, USA). IL-1β cytokine titers were measured in the serum by ELISA procedure as described in previous section.

### Statistical Analysis

Statistical analysis was performed with Prism 6.01 (Graphpad, La Jolla, CA, USA) software. Statistical significance was determined using one-way analysis of variance followed by Tukey’s and Bunnett’s multiple comparison test. The *P*-values ≤0.05 were considered statistically significant.

## Results

### Characterization of NP Formulations

**Figure 1:**
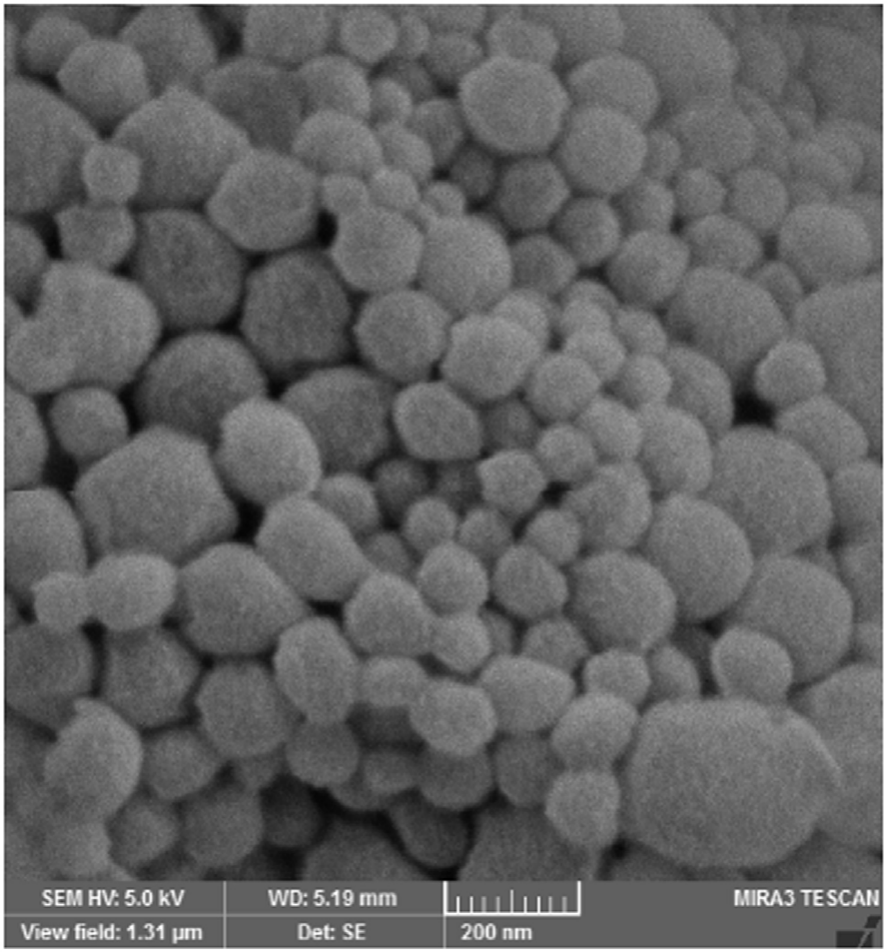
Scanning electron microscopy image of poly(lactic-co-glycolic) acid/polyethylenimine structure.

**Figure 2:**
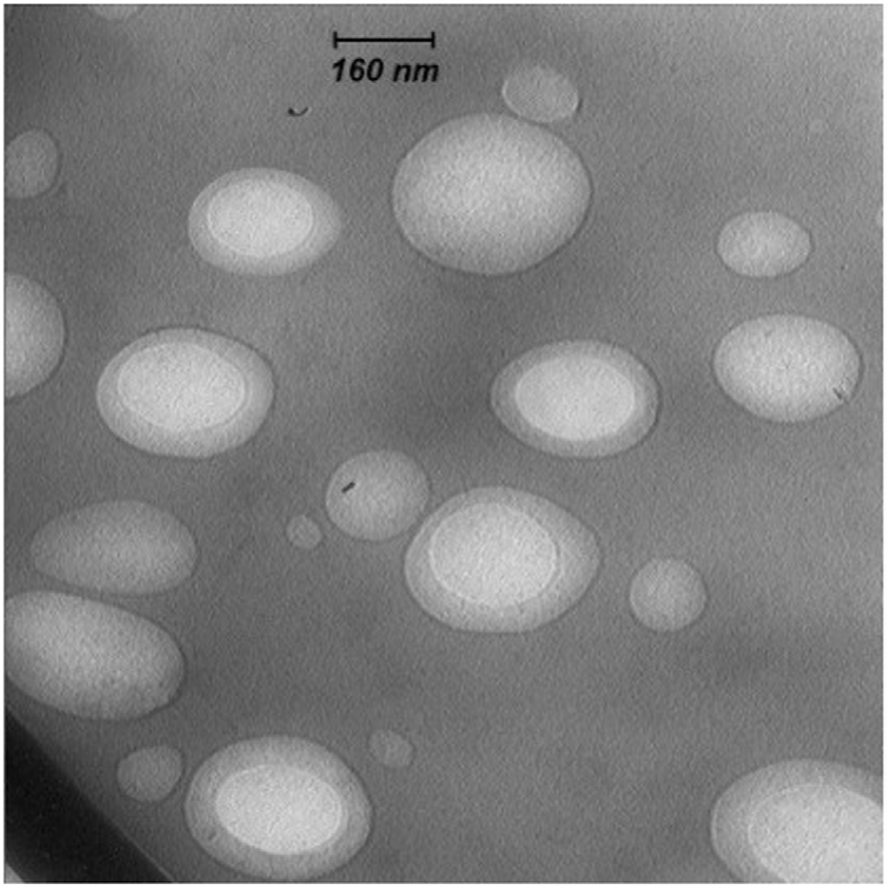
Transmission electron microscopy image of poly(lactic-co-glycolic) acid/polyethylenimine core–shell structure.

The mean diameters of OVA-loaded, MPLA-loaded, and R848-loaded NPs were 208, 225, and 221 nm, respectively. The zeta potential was negative for NPs varying from ~−13 to −15 mV. All cationic PLGA-PEI/ODN polyplexes, at all C/P ratios, had effective diameters of less than 180 nm. Zeta potential was in the range of 22–25 mV for all polyplexes (Table 2). The SEM image of PLGA(MPLA)-PEI/ODN polyplex showed smooth surface and spherical shape (Figure 1). Moreover, a core–shell particle structure is envisaged with a PLGA core containing MPLA and a PEI coating. The core–shell structure of the PLGA/PEI NPs was confirmed by TEM analysis (Figure 2). Slight size changes were observed following complexes of the PLGA/PEI NPs with ODN as reported in other studies (25).

**Table 2:** Characterization of MPLA-loaded, R848-loaded, and OVA-loaded PLGA/PEI core–shell co-polymer formulations containing CpG ODN.

Formulation	C/P ratio	Size (nm)	Zeta potential (mV)
PLGA (MPLA) NPs	–	225 ± 15	−15 ± 0.7
PLGA(MPLA)-PEI/ODN polyplex	2	180 ± 5	23 ± 0.7
PLGA(MPLA)-PEI/ODN polyplex	4	140 ± 5	25 ± 0.6
PLGA(MPLA)-PEI/ODN polyplex	6	128 ± 2	22 ± 0.2
PLGA (R848) NPs	–	221 ± 2	−15 ± 0.7
PLGA(R848)-PEI/ODN polyplex	2	135 ± 7	20 ± 0.3
PLGA(R848)-PEI/ODN polyplex	4	130 ± 15	25 ± 0.8
PLGA(R848)-PEI/ODN polyplex	6	125 ± 4	26 ± 0.5
PLGA (OVA) NPs	–	208 ± 7	−12.9 ± 2.2

*CpG ODN, cytosine–phosphorothioate–guanine oligodeoxynucleotide; MPLA, monophosphoryl lipid A; NPs, nanoparticles; OVA, ovalbumin; PEI, polyethylenimine; PLGA, poly(lactic-co-glycolic) acid; R848, resiquimod*.

### Agarose Gel Retardation Assay

**Figure 3:**
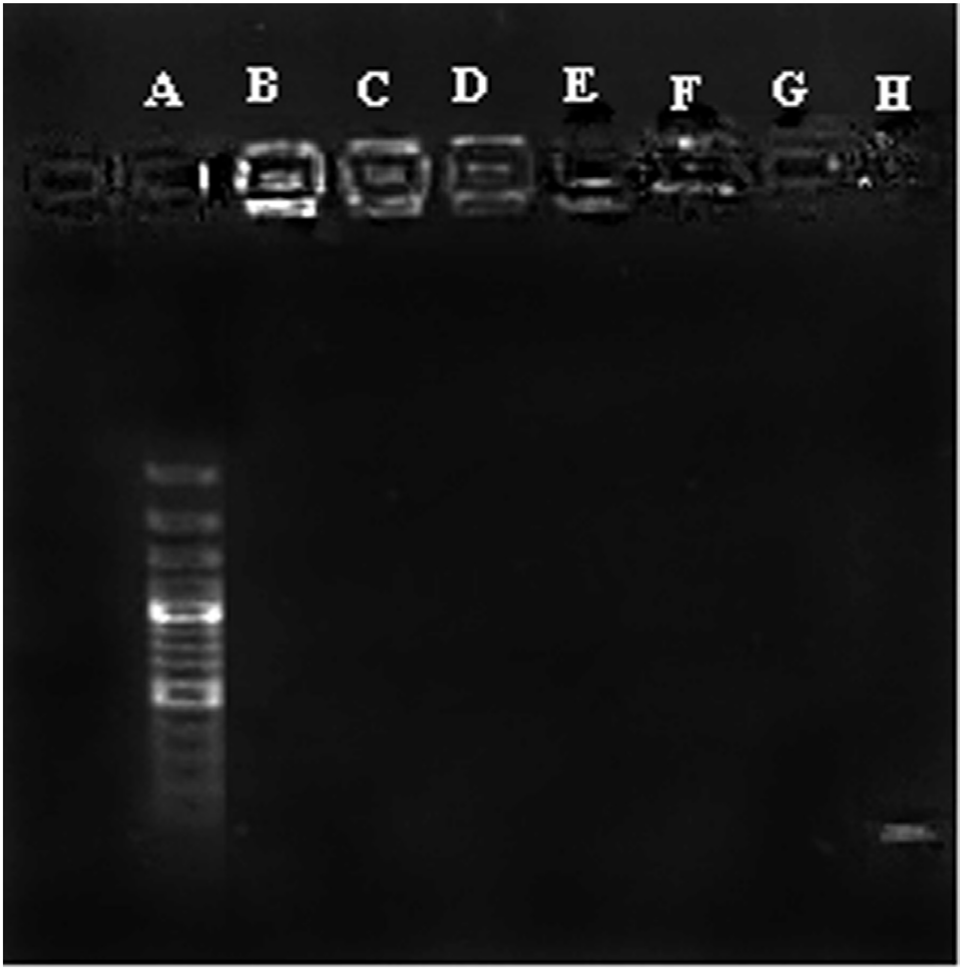
Agarose gel retardation assay of cationic poly(lactic-co-glycolic) acid (PLGA)/polyethylenimine (PEI) nanoparticles. A: Ladder, B–D: PLGA/PEI CP 2-4-6, E–G: PEI CP 2-4-6, H: cytosine–phosphorothioate–guanine oligodeoxynucleotide alone.

Condensation of CpG ODN into nano-sized particles is an essential requirement for efficient delivery of ODN to the cells. Condensation of CpG ODN by PLGA/PEI was evaluated by agarose gel retardation assay. Results indicated that the complex was able to efficiently condense ODN at all C/P ratios (Figure 3).

### *In Vitro* Release Study

**Figure 4:**
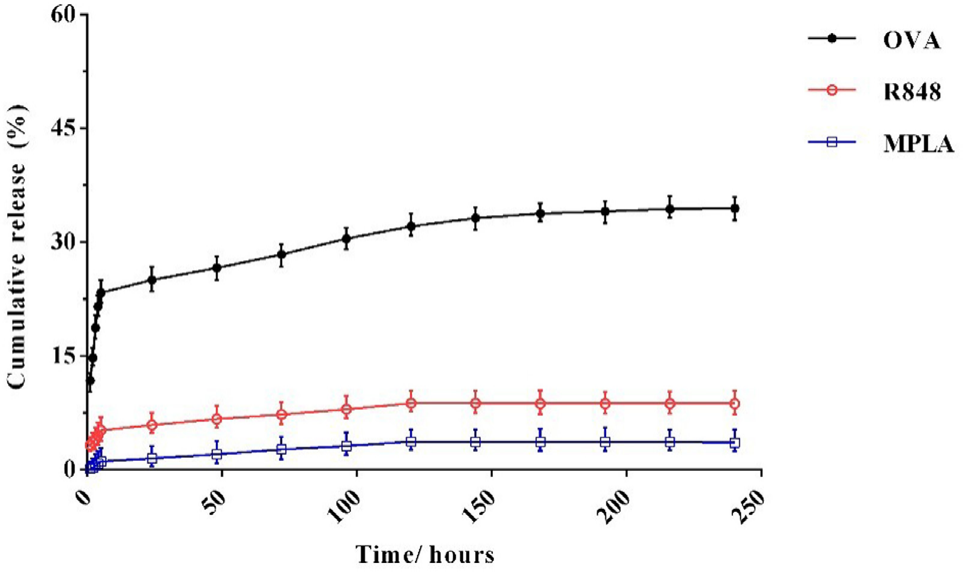
*In vitro* release profile of monophosphoryl lipid A, resiquimod, and ovalbumin from poly(lactic-co-glycolic) acid nanoparticles over the time. The experiment was performed in PBS in 37°C. Indicated values are mean (±SD) of at least three experiments.

**Figure 5:**
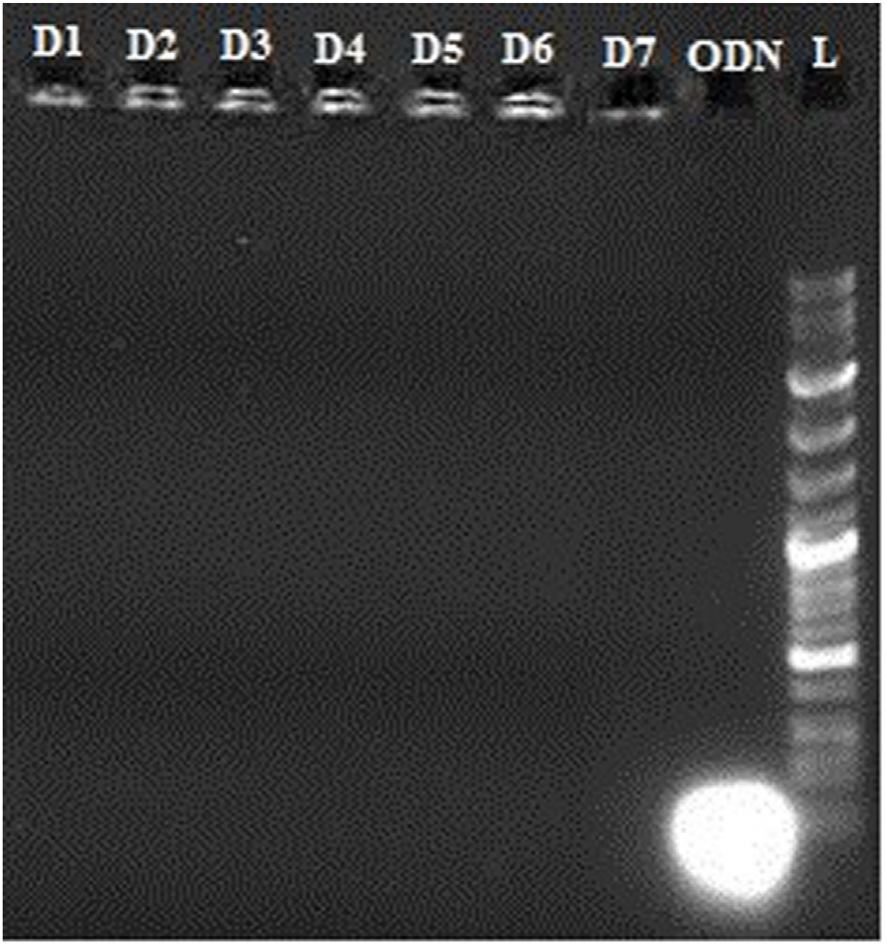
Stability of vector–cytosine–phosphorothioate–guanine oligodeoxynucleotide conjugation by gel electrophoresis after 7 days. L: Ladder 100k, ODN: CpG ODN, D1–7: Days 1–7.

*In vitro* release of OVA, R848, and MPLA from PLGA NPs in PBS at 37°C was evaluated over a period of 10 days. As shown in Figure 5, about 23% of the total amount of OVA is released from the NPs during the first 5 h. This initial release of OVA is attributed to antigen located near the NPs external surface. This phenomenon is consistent with high amount of OVA reported to reside on the external surface and/or in the pores connected to the surface of PLGA particles. The release profile of the MPLA and R848 was found to be similar to that of OVA (Figure 4). Only a small amount of MPLA (6%) and R848 (9%) was released from the PLGA NPs after 10 days of incubation in PBS at 37°C, and after 7 days, a plateau in release of these adjuvants was reached. Also as shown in Figure 5, stable conjugation between the CpG ODN and vector formed as after 7 days it remained condensed in agarose gel electrophoresis.

### EE% of PLGA (OVA), PLGA (R848), and PLGA (MPLA) NPs

The EE% of NPs loaded with OVA, R848, or MPLA were 96 ± 3, 98 ± 2, and 60 ± 2.2%, respectively (Table 3).

**Table 3:** The encapsulation efficiency of different formulations.

Formulation	Encapsulation efficiency (%)	
	R848, OVA (UV–Vis)	MPLA (fluorimetry)
PLGA (OVA)	96 ± 3	–
PLGA (R848)	98 ± 2	–
PLGA (MPLA)	–	60 ± 2.2

*MPLA, monophosphoryl lipid A; OVA, ovalbumin; PLGA, poly(lactic-co-glycolic) acid; R848, resiquimod*.

### Evaluation of Cytotoxicity Using MTT Assay

**Figure 6:**
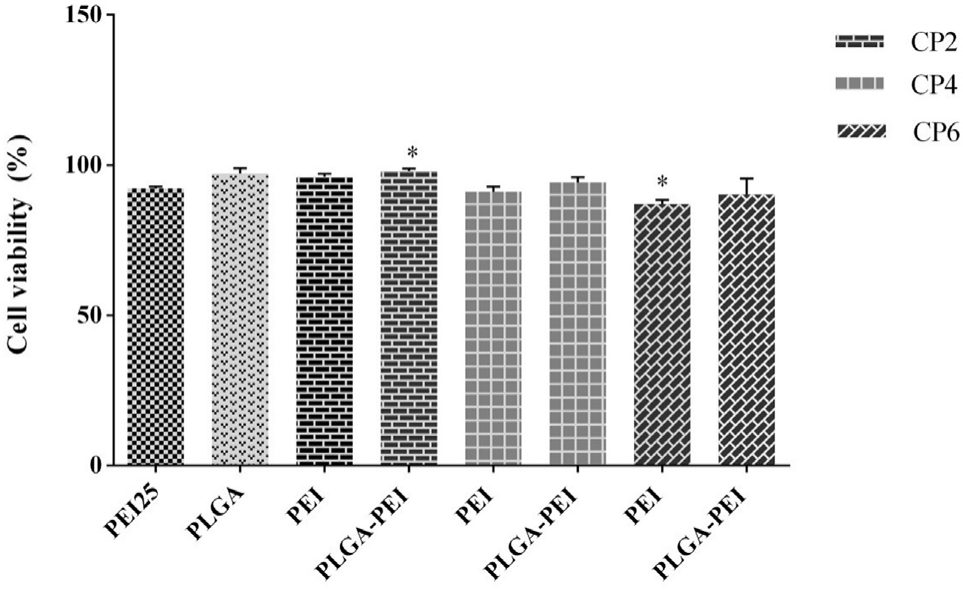
Comparison of cellular viability of polyethylenimine (PEI) 25 kDa (C/P 0.8) as control with PEI and cationic poly(lactic-co-glycolic) acid/PEI complexed with cytosine–phosphorothioate–guanine oligodeoxynucleotide up to C/P 6 in J774 cells. Cells were treated for 48 h under the condition used in uptake assay, and then cell viability was assessed using MTT. The results are reported as mean ± SD, *n* = 3.

*In vitro* cytotoxicity of PEI and PLGA/PEI at C/P 2, 4, and 6 were evaluated in J774 cells. PEI 25 kDa at C/P 0.8 was used as positive control. PEI and PLGA/PEI at C/P 2 and 4 did not exhibit pronounced cytotoxicity and only PEI and PLGA/PEI at C/P 6 showed moderate cytotoxicity in comparison with PEI 25 kDa. However, the results indicated a viability of more than 95% at all C/P ratios tested (Figure 6). We should point out that the only material for which cytotoxicity was expected was PEI. As reported, no cytotoxicity test was performed for either MPLA or R848 as these adjuvants were used at very low doses in our study (14, 26).

### Uptake Study on J774 Cells Treated with CpG ODN Complexed Cationic NPs

**Figure 7:**
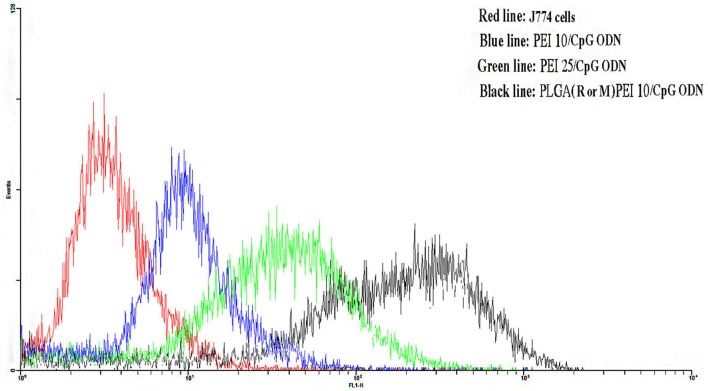
Flow cytometry histogram showing the uptake of polyethylenimine (PEI) 10/cytosine–phosphorothioate–guanine oligodeoxynucleotide (CpG ODN, blue line) and PEI25/CpG ODN (green line) as positive control groups and poly(lactic-co-glycolic) acid (R or M)/PEI10/CpG ODN (black line) in J774 cells after 48 h incubation. J774 cells were used as negative control group. The R or M stands for either R848 or monophosphoryl lipid A.

The uptake efficiency of FITC-labeled NPs prepared from PLGA (MPLA or R848)/PEI complexed with CpG ODN was determined in J774 cells by flow cytometry. As shown in Figure 7, PLGA (MPLA or R848)/PEI NPs exhibited significantly more uptake than PEI10-CpG ODN and PEI25-CpG ODN as positive control groups at mass ratio tested (C/P 2).

### *In Vivo* Induction of Immune Responses by Various NP Formulations

**Figure 8:**
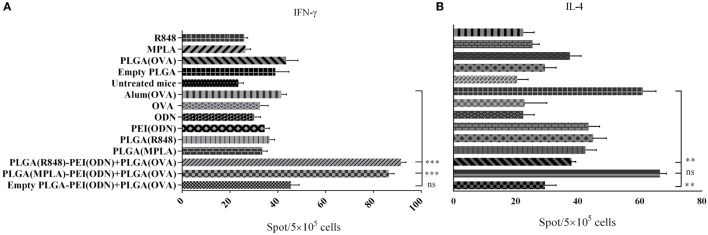
The effect of different poly(lactic-co-glycolic) acid (PLGA)/polyethylenimine (PEI) formulations harboring dual toll-like receptor (TLR) agonists as adjuvant in inducing cellular immune responses evaluated by measuring the number of IFN-γ and IL-4 producing splenocytes using ELISpot assay. BALB/c mice (three mice per group) were immunized twice (at days 0 and 21) with different dual core–shell adjuvant and PLGA [ovalbumin (OVA)] NP formulations. On day 7 post booster, mice from each group were sacrificed, and their splenocytes were stimulated with OVA protein for 24 h. IFN-γ and IL-4 release from splenocytes induced by different PLGA/PEI adjuvant formulations were determined using ELISpot assay. The data indicate the mean ± SEM (*n* = 3) **P ≤ 0.01, ***P ≤ 0.001 (one-way analysis of variance). The asterisks located on top of the lines showed comparison of significance between the main (dual TLRs agonist) and control (OVA + aluminum hydroxide) group.

To investigate how immune responses is influenced by administration of different tripartite NPs formulation *in vivo*, we used OVA as a model antigen for encapsulation into PLGA NPs along with either PLGA (MPLA)-PEI/(CpG ODN) or PLGA (R848)-PEI/(CpG ODN) NPs. These formulations were injected S.C. either alone or in combination with PEI/CpG ODN into the base of tail of BALB/c mice. Single TLR agonists, either encapsulated in PLGA or in soluble forms, were also used as control. One week after the second immunization, splenocytes were removed and after 24 h of *in vitro* restimulation with antigen, spleen cells were assessed for IL-4 and IFN-γ secretion by ELISpot assay. As shown in Figure 8, in immunized animals with PLGA (R848)-PEI/(CpG ODN) NPs, IL-4 production significantly diminished while IFN-γ production increased to a level higher than the control group (OVA + Alum). Also, there was a significant different (*P* < 0.05) in secretion of IFN-γ between immunized animals with PLGA (MPLA)-PEI/(CpG ODN) and control group (OVA + Alum). We also observed significant differences in IFN-γ secretion between animal immunized with dual TLR agonist compared to single TLR agonists. In other words, when MPLA as TLR4 ligand or R848 as TLR7/8 agonist is co-delivered with CpG ODN as TLR9 ligand using PLGA/PEI NPs, a significant increase in IFN-γ secretion can be observed in comparison to single TLR ligands (encapsulated in PLGA) and alum-adjuvanted OVA group. As for the IL-4 secretion, while a significant effect of using dual TLR ligands (MPLA + CpG ODN) is observed as compared to PLGA (R848)-PEI/(CpG ODN) NPs as well as PLGA-PEI/(CpG ODN) group, no significant differences in IL-4 secretion can be observed in comparison to alum-adjuvanted OVA group.

**Figure 9:**
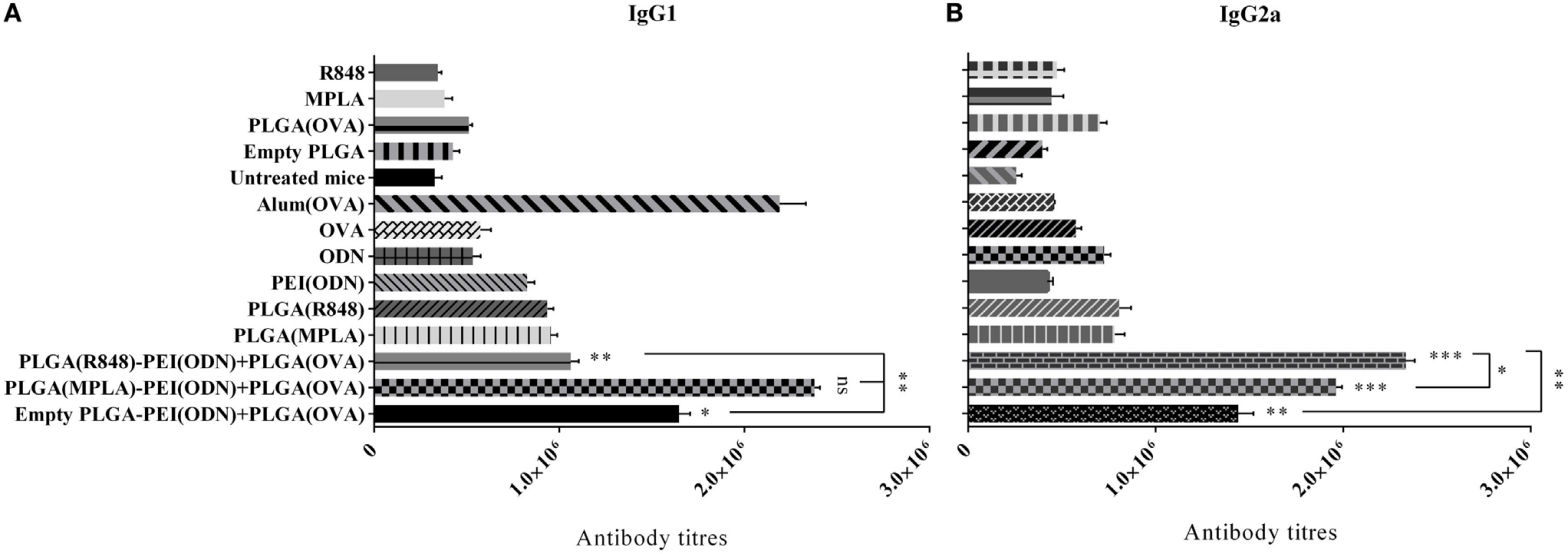
The effect of co-administration of poly(lactic-co-glycolic) acid (PLG)A [ovalbumin (OVA)] nanoparticles along with PLGA [monophosphoryl lipid A (MPLA) or resiquimod (R848)]/polyethylenimine core–shell complexed with cytosine–phosphorothioate–guanine oligodeoxynucleotide (CpG ODN) on IgG1 and IgG2a isotype responses in immunized BALB/c mice. The IgG1 and IgG2a antibody titers in the serum samples were measured by enzyme-linked immunosorbent assay. IgG1 **(A)** and IgG2a **(B)** isotype antibody titers 4 weeks after primary immunization (mean ± SD with three mice per treatment group). ***P < 0.001, **P < 0.01, and *P < 0.05 (one-way analysis of variance). The asterisks located on top of error bars showed comparison of significance between the main [dual toll-like receptor (TLR) agonist] and control (OVA + aluminum hydroxide) group and the asterisks located on top of the lines showed comparison between the main (dual TLR agonist) groups.

To further characterize the immune response generated after immunization of mice with various nanoparticulate adjuvant formulations, we measured IgG1 and IgG2a antibody isotype titers in the serum of immunized animals by ELISA one week after the second immunization. As indicated in Figure 9, a strong antibody response in terms of IgG1 and IgG2a isotypes was detected after *in vivo* administration of dual TLR agonist formulations (MPLA or R848 inside and CpG ODN outside PLGA/PEI NPs). Like cytokine detection, a high level of both IgG1 and IgG2a was observed after immunization of animals with dual TLR agonists containing PLGA (MPLA)-PEI/(CpG ODN) (Figure 9). A high titer of IgG2a, which correspond to Th1 immune response, was also observed when dual TLR7/8 and TLR9 agonists (R848 + CpG ODN) were delivered by PLGA/PEI NPs. The highest level of IgG1 which correspond to Th2 immune response was achieved after *in vivo* administration of dual TLR4 and TLR9 agonists (MPLA + CpG ODN) co-delivered by PLGA/PEI NPs. This response was comparable to positive control group (alum-adjuvanted OVA).

**Figure 10:**
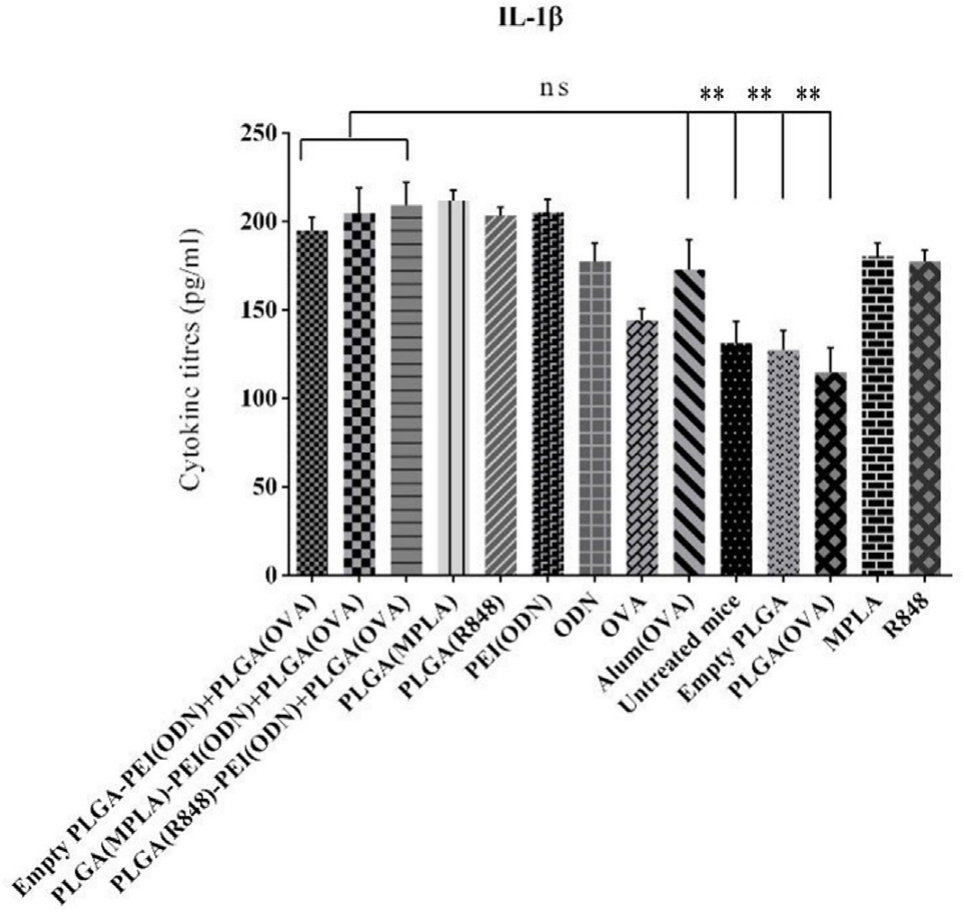
Detection of IL-1β level in the serum in BALB/c mice immunized with various toll-like receptor (TLR) agonists adjuvant formulations along with poly(lactic-co-glycolic) acid (PLGA) [ovalbumin (OVA)] NPs. Serum IL-1β level was determined using enzyme-linked immunosorbent assay. There was no significant difference in IL-1β secretion between different adjuvant formulations in comparison with positive control (OVA + aluminum hydroxide). The asterisks located on top of the lines showed comparison of significance between the main (dual TLRs agonist) and naive (untreated mice) as well as mice immunized with empty PLGA and PLGA (OVA). The data are indicated the mean ± SD (*n* = 3).

We also measured the level of IL-1β as a main pro-inflammatory cytokine important in initiating the innate immune responses, in the serum of immunized animals by ELISA one week after the second immunization. As indicated in Figure 10. A steady state increased level of IL-1β detected in all adjuvant immunized animals as compared to naive animals (untreated mice) points to the activation of innate responses, which is a pre-requisite of any effective acquired immune responses generated by infection or vaccination.

The relative order of effectiveness of various adjuvant formulations used in this study in inducing IFN-γ, IL-4, IgG1, and IgG2a secretion is summarized in Table 4.

**Table 4:** Summary of *in vivo* antibody isotype as well as cytokine secretions elicited by various PLGA/PEI adjuvant formulations.

Group	Delivery systems	Alum (μg)	OVA (μg)	R848 (μg)	MPLA (μg)	CpG ODN (μg)	IgG1	IgG2a	IFN-γ	IL-4
1	PEI, PLGA	–	10	–	–	10	+++	++	++	++
2	PEI, PLGA	–	10	–	3	10	++++	+++	+++	++++
3	PEI, PLGA	–	10	4	–	10	++	++++	++++	++
4	PLGA	–	–	–	3	–	++	+	+	++
5	PLGA	–	–	4	–	–	++	+	+	++
6	PEI	–	–	–	–	10	++	+	+	++
7	–	–	–	–	–	10	+	+	+	+
8	–	–	10	–	–	–	+	+	+	+
9	–	100	10				++++	+	++	+++
10	–	–	–	–	–	–	+	+	+	+
11	PLGA	–	–	–	–	–	+	+	+	++
12	PLGA	–	10	–	–	–	+	+	+	+
13	–	–	–	–	3	–	+	+	+	+
14	–	–	–	4	–	–	+	+	+	+

*The level of IgG isotypes or cytokine levels in descending order is indicated by ++++, +++, ++, and +. Highest level illustrated by ++++ and lowest level +*.

*Alum, aluminum hydroxide; CpG ODN, cytosine–phosphorothioate–guanine oligodeoxynucleotide; MPLA, monophosphoryl lipid A; OVA, ovalbumin; PEI, polyethylenimine; PLGA, poly(lactic-co-glycolic) acid*.

## Discussion

Recently, we have described a novel delivery vehicle for TLR9 ligand (CpG ODN) based on single-walled carbon nanotube functionalized with PEI and demonstrated its efficacy in induction of Th1/Th2 immune responses in mice (27). Given the pivotal role of PRRs and specially TLRs in initiating and tuning of immune responses in the context of vaccine-adjuvant development, the present study was conducted to synthesize and evaluate a PLGA NP-based adjuvant delivery platform using multiple TLRs (TLR4, TLR7/8, and TLR9) agonists. MPLA as TLR4 agonist and R848 as TLR7/8 agonist were encapsulated in PLGA NPs. Subsequently, CpG ODN as TLR9 agonist was physically linked to the core–shell of PLGA (MPLA) or PLGA (R848) using PEI to form a hybrid PLGA NPs containing dual TLR agonists (MPLA + CpG ODN or R848 + CpG ODN). *In vivo* immunization of these dual adjuvant formulations along with OVA-encapsulated PLGA NPs as antigen in mice elicited efficient Th1-skewed cytokine (IFN-γ) and antibody (IgG2a)-mediated responses compared to single TLR agonist (encapsulated in PLGA) and OVA encapsulated PLGA, or OVA with or without alum. Targeting antigens along with TLR agonists encapsulated in PLGA NPs to APCs is a promising approach for generating potent Th1 polarizing immune responses that can be potentially useful in immunotherapy of cancer and intracellular pathogens (28). In this respect, activation of TLR4 by LPS and TLR9 by CpG ODN induces strong Th1 immune responses through the secretion of IL-12p70 (29). Furthermore, it has been reported that, stimulation of IFN-α secretion by TLR3, TLR4, TLR7, and TLR9 is an important driving force of TLR-mediated Th1 immune responses (30). Also, it was shown that multiple TLR activation (TLR3, TLR4, TLR7, TLR8, and TLR9) improved and sustained Th1 immune responses, through the enhancement of IL-12 and IL-23 production in both human and mouse DCs (11). Additionally, we observed a significant Th2 skewing cytokine (IL-4) and antibody (IgG1) responses when the combination of TLR4 and TLR9 agonists along with OVA was delivered by PLGA NPs, suggesting the effects of these TLR agonists on activation of both Th1 and Th2 mediated immune responses. The observed Th2 responses seen in our study were significantly higher than OVA with or without encapsulation in PLGA and comparable to immunization with combination of OVA plus alum, both of them considered to be potent Th2 inducers (1). PEI used in the construction of hybrid PLGA NPs in our study forms a polyplex with CpG ODN (as a shell) on the core of PLGA NPs resulting in enhanced immunogenicity of the polyplexes due to increased antigen uptake by APCs, improved trafficking of DCs to draining lymph nodes, and induction of Th1/Th2 cytokine profiles (31). Potential weakness of PEI is that high molecular PEI is toxic to cells due to plasma membrane and/or lysosomal damage by the proton-sponge effects (32, 33). Thus, PEI with optimal molecular weight (10 kDa) which we used in this study is considered to be safe and effective in the context of adjuvant development and can deliver the adjuvant cargo to the phagosome compartment of APCs efficiently (27).

Arranging TLR ligands on or inside PLGA NPs, which are in the size range of viruses and bacteria, created biomimetic platforms for efficient interactions with APCs, inducing synergistic antibody as well as T cell-mediated immune responses *in vitro* and *in vivo* which is required for efficient vaccine immunogenicity (34).

Given the size (180 ± 5 and 135 ± 7 nm) of the vehicles constructed in our study which are in the size range of pathogens like viruses and small bacteria and the presence of dual TLR agonists on and inside the NPs, this could be considered as an artificial biomimetic approach to resemble pathogen-like molecules harboring artificial PAMPs. During natural infection, most pathogens present multiple PAMPs to initiate the innate immune responses through multiple stimulation of PRRs, resulting in activation of innate and adoptive immune responses (4, 30). Therefore, pathogen-mimicking NPs hold great potential as vaccine and adjuvant delivery system due to their ability to induce and enhance cytokine secretion and recruitment of professional immune cells at the injection site as well as stronger humoral and cellular immune responses *in vivo* (35, 36). The magnitude and direction of vaccine-adjuvant responses generated by successful vaccination can serve as an ideal model for design and development of novel nanoparticulate vaccine-adjuvant combination. Despite the shortcoming of many traditional vaccines, yellow fever vaccine 17D is one of the safest and efficacious live attenuated vaccines ever developed to date, still controlling outbreaks in modern day (37). Its efficacy likely results from activation of multiple TLRs (TLR2, TLR7, TLR8, and TLR9) on plasmacytoid and myeloid DCs, characterized by a mixed Th1/Th2 cytokine profile and antigen-specific CD8^+^ T cells (38), suggesting that synergistic activation of multiple TLRs is a crucial step in promoting effective vaccine immunogenicity (30). Therefore, given the importance of TLRs in linking innate and adaptive immunity by both inducing APC maturation, Th cell activation, and attenuating suppressor functions of regulatory T cells (39), the data presented here provide additional credit to a novel biomimetic approach for induction of robust Th1/Th2 immune responses generated by PLGA-based NPs containing multiple TLRs agonist to enhance the vaccine-adjuvant immunogenicity. Further research is necessary to reveal whether the NP formulation strategies like this with reduced dose of antigen-adjuvant and the increased effect observed between TLR agonists will still be effective and safe in implementing and orchestrating the optimal and effective *in vivo* vaccine responses.

## Ethics Statement

Animal care and all animal experiments were performed after approval of the Institutional Ethical Committee and Research Advisory Committee of Mashhad University of Medical Sciences and Ferdowsi University of Mashhad based on the national guidelines from Ministry of Health and Medical Education of Iran, adopted from the 86/609/EEC Directives of European Community. Mice were housed in animal cages before performing the study and were kept with free access to water and food.

## Author Contributions

ME performed the experiments, analyzed data, assisted in writing, revision, and edited the paper; MH assisted in analyzing data and edition of the paper; MM, GH, and KA provided reagents; MR provided reagents, designed experiments, assisted in analyzing data, and edition of the paper; and AH conceived the idea, designed experiments, provided reagents, analyzed data, wrote, edited, and revised the entire manuscript.

## Conflict of Interest Statement

The authors declare that the research was conducted in the absence of any commercial or financial relationships that could be construed as a potential conflict of interest.
